# Taking advantage of opportunistically collected historical occurrence data to detect responses to climate change: The case of temperature and Iberian dung beetles

**DOI:** 10.1002/ece3.10674

**Published:** 2023-12-06

**Authors:** Jorge M. Lobo, Mario Mingarro, Martin Godefroid, Emilio García‐Roselló

**Affiliations:** ^1^ Departamento de Biogeografía y Cambio Global Museo Nacional de Ciencias Naturales–CSIC Madrid Spain; ^2^ Departamento de Informática University of Vigo Ourense Spain

**Keywords:** collection bias, dung beetles, Iberian Peninsula, spatio‐thermal patterns, species decline, temporal trends

## Abstract

This study introduces a novel approach to leverage high‐resolution historical climate data and opportunistically collected historical species occurrence data for detecting adaptive responses to global change. We applied this procedure to the temperature data and the most comprehensive Iberian dataset of dung beetle occurrences as an illustrative example. To understand how populations of different species are responding, we devised a procedure that compares the temporal trend of spatial and temperature variables at the locations and times of all the occurrence data collection (overall trend) with the specific temporal trends among the occurrences of each species. The prevalence of various species responses is linked to life history or taxonomic characteristics, enabling the identification of key factors influencing the propensity to experience different effects from climate change. Our findings suggest that nearly half of the Iberian dung beetle species may be adversely affected by temperature increases, with a geographic shift being the most common response. The results generated through the proposed methodology should be regarded as preliminary information, serving to formulate hypotheses about the diverse responses of species to climate change and aiding in the selection of candidate species capable of coping with challenges posed by changing temperatures.

## Graphical abstract

A novel approach for taking advantage of historical occurrence data to detect adaptive responses to climate change is presented. Our findings suggest that approximately half of the Iberian dung beetle species may experience negative effects from temperature increases and that the spatial response will be the most frequent one.

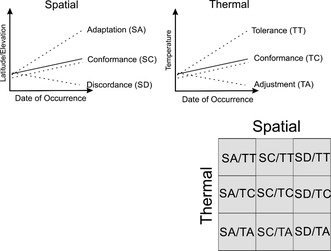

## 1 INTRODUCTION

The increasing availability of digital data associated with the collection of organisms sampled over 300 years presents a major opportunity to illustrate patterns of global biodiversity and understand the processes that generated them. Although the first efforts to convert biodiversity information into a computer‐readable format date back to the 1990s, the aggregation of digital data into freely accessible biodiversity databases has increased exponentially over the course of the 21st century (Nelson & Ellis, 2018). Unsurprisingly, the biodiversity data compiled by different and uncoordinated initiatives (Feng et al., 2022) are almost always characterized by the pervasive existence of taxonomical and geographical biases and shortcomings (see, for example, Hortal et al., 2015; Hughes et al., 2021; Meyer et al., 2016; or Larsen & Shirey, 2021). These drawbacks, inherent to opportunistically collected historical occurrence data, may limit but not invalidate the use of such information for scientific or conservation purposes (Grand et al., 2007; Isaac et al., 2014; Park, Lyra, et al., 2023; Park, Xie, et al., 2023; van Strien et al., 2013). Different approaches to filtering and processing information from biodiversity databases have allowed taking advantage of this unprecedented source of information to propose explanatory hypotheses about the spatiotemporal distributions of organisms (Belitz et al., 2020; Di Cecco et al., 2023; García‐Roselló et al., 2015; Heberling et al., 2021; Isaac et al., 2014; Lajeunesse & Fourcade, 2023; Pagel et al., 2014).

Understanding the responses of ecological communities to climate change is a critical aspect of conservation planning in a rapidly changing world. Two main scenarios of adaptive responses, which are not mutually exclusive, have been identified for a wide variety of plant and animal species. First, populations can adapt to new climatic conditions in the regions they currently inhabit through physiological, genetic, or behavioral adjustments (or a combination of all these) (Parmesan, 2006). Among these mechanisms, one of the most widely observed is the temporal shift of climatically constrained life‐history events to more suitable times of the year (i.e., phenological adaptation; Parmesan & Yohe, 2003) through phenotypic plasticity (Sauve et al., 2019; Whitman & Ananthakrishnan, 2009) or micro‐evolutionary changes (i.e., heritable genetic changes; Anderson et al., 2012). Second, when populations are unable to adjust their physiology or behavior to new climatic conditions, they may spatially track suitable climate conditions by shifting their geographic range (“spatial adaptation” sensu Hengeveld and Hemerik (2002)). In many species, both recent range shifts, mainly towards higher latitudes and/or elevations, and range contraction at the southern edges of the distributions have been observed (Battisti et al., 2005; Hickling et al., 2006; Parmesan et al., 1999; Parmesan & Yohe, 2003). As an example, according to Parmesan and Yohe (2003), in the Northern Hemisphere, a set of species range boundaries shifted, on average, 6.1 km per decade northwards and/or 6.1 m per decade upwards.

The different adaptive responses to climate change depend mainly on species' life‐history traits (reproduction, phenology, dispersal abilities, physiology; Estrada et al., 2016) and additional factors such as landscape composition, human activities, and biotic interactions. For example, biotic interactions may be particularly important when the fate of a particular species is intimately linked to the response of interacting species such as host plants, predators, prey, competitors, and parasites (Visser & Gienapp, 2019). In some cases, a distribution shift or phenological change may not be successful if the interacting species do not respond in the same spatial or temporal direction (Donoso et al., 2016). When biotic and abiotic limiting factors or inherent behavioral, genetic or physiological constraints are too influential, some populations or even whole species may be unable to shift their geographical distributions and/or adjust their phenology to adapt to new climatic conditions. Those species that are unable to adapt may be at risk of a significant population decline, ultimately leading to the extinction of local populations or, in the worst‐case scenario, the complete extinction of the species. Therefore, identifying species at risk of extinction that will be capable of adaptive adjustments is especially important for the design of conservation plans.

In this context, insects deserve particular attention. Insects are providers of many crucial ecosystem services (e.g., pollination, nutrient cycling, decomposition or biological control), so their responses to global change have profound implications for ecosystem health and human well‐being. Therefore, the impact of global change on insects has attracted the attention of the scientific community (see Harvey et al., 2023; Ursul et al., 2023). Because most insects are ectothermic (i.e., they generally cannot produce their own body heat) and poikilothermic (i.e., in which internal temperature varies considerably), they are considered to be particularly sensitive to temperature variations. This makes them promising biological indicators of the potential impact of ongoing climate change on biological communities. Many recent studies suggest that substantial and widespread declines in insect populations are now occurring in various parts of the world due to habitat loss, pollution and climatic change (Halsch et al., 2021; Outhwaite et al., 2022; Soroye et al., 2020; Wagner et al., 2021). Although it is difficult to distinguish the relative contribution of climate change in these declines, it is assumed that it may cause deleterious impacts on many species and that negative synergies occur between habitat degradation and climate change (Mingarro et al., 2021; Outhwaite et al., 2022). As a consequence, there is growing interest in understanding and anticipating the impact of climate change on insects. There is a need to prioritize conservation efforts for species that may be unable to develop an effective adaptive response to global change. Ideal studies of the responses of species to climate change have been based on long‐term standardized monitoring data. These studies may use temporal data from a single locality on key life‐history events (phenological studies; e.g., Mingarro et al., 2021; Ovaskainen et al., 2020), or they may use time series of distribution data sampled repeatedly across the entire range of a species or at its distribution limits (range shift studies; e.g., Tingley & Beissinger, 2009). Unfortunately, ideal long‐term standardized monitoring data are available only for a negligible number of species. However, a huge amount of opportunistically collected historical occurrence data exists, which constitutes an underutilized but promising resource that can provide crucial information on species responses to environmental changes (Belitz et al., 2020; Di Cecco et al., 2023; Isaac et al., 2014; Lajeunesse & Fourcade, 2023; Pagel et al., 2014).

This study introduces an approach that harnesses high‐resolution historical climate data and opportunistically collected species occurrence records to explore their responses to climate change. To illustrate our methodology, we have used temperature data and information on a specific group of insects, namely dung beetles, within the context of the Iberian Peninsula. Temperature was chosen due to its well‐established significance as a fundamental factor influencing species distributions (Antão et al., 2020). However, it is essential to emphasize that our approach is adaptable and can be applied with various climatic variables. By combining climatic and occurrence data, we aim to provide an initial assessment of the relative importance of different types of species' responses to temporal variations in temperature. We first describe the biological rationale of the proposed procedure, describing the main hypotheses related to the different potential responses of species to temperature change. The most plausible hypothesis is then selected by examining the historical trend in the temperatures prevailing in the locations and times at which the occurrences were collected. The prevalence of different species responses are subsequently related to life history or taxonomic characteristics to hypothesize key factors shaping the propensity to experience different effects from climate change.

## 2 CONCEPTUAL FRAMEWORK

The present procedure aims to derive possible explanatory hypotheses about the effects of temperature changes on species assemblages using simple historical species occurrence datasets that include, at a minimum, the sampling date (month and year), the sampling geographic location (latitude and longitude), and elevation. The data are assumed to represent species incidences along a temporal sequence of increases in temperature. Thus, we must first check that the temperature of the region under study has increased significantly during the time to which the species data pertain. If this is the case, the progressive increase in the date of the incidence records can be used as a surrogate of the temperature increase (i.e. the closer the date is to the present, the warmer the temperature will be).

In a second phase, the procedure will seek to estimate the overall trend of the temporal variation in temperature for the considered geographical region. This overall trend (OT) is determined by taking into account the complete set of records for all the species of the considered target group. OT thus represents the average temperature for the data of the studied species group during the sampling period (Box 1). Unfortunately, this OT may be affected by the spatially and temporally biases and shortcomings that are common in biodiversity databases (García‐Roselló et al., 2023; Lobo et al., 2007). Therefore, the interpretation of an overall trend must be done with caution. For example, it may occur that more recent records were collected at higher elevations due to collection biases or preferences or that low elevation areas were overlooked by collectors due to disturbances in land use. Regardless of the source of bias, the overall trend reflects the biases and/or general patterns of the whole dataset, although the intensity and direction of these biases will depend on the study group. Be that as it may, those uncertainties and possible biases related to the causes of the temporal response shown by this overall trend prevent us from interpreting it as reliable information about the main adaptive response of the considered assemblage. Despite these limitations, we propose using the OT as a neutral baseline for comparison with the spatiotemporal response of each individual species in the dataset.

BOX 1 **Hypothetical temporal tendencies of the species occurrence data across spatial and temperature gradients.**

Relationships between the spatial locations of species occurrences (Latitude/Elevation) or the temperature of the observations (Temperature) and the time at which the observations were collected (Date of Occurrence). The continuous lines represent the general pattern of the complete set of records of the considered target group (i.e., the overall trend or OT), while the dashed lines represent the hypothetical relationships of a specific species.

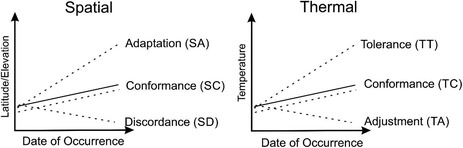

Species spatial data may show a positive (spatial adaptation or SA) or negative (spatial discordance or SD) temporal slope significantly different from the slope coming from the overall trend. In contrast, these occurrences can show a temporal variation in their spatial location similar to the one represented by the overall trend (spatial conformance or SC). Thermal response over time may also show a positive (thermal tolerance or TT) or negative (thermal adjustment or TA) temporal response to temperature that is significantly different from the response shown by the overall trend. Here, too, these temporal temperature trends can be similar (thermal conformance or TC) to those experienced by the overall trend.

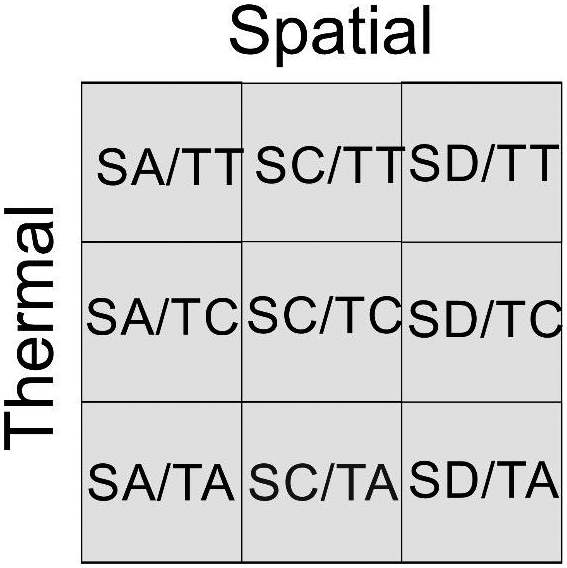

The combination of these spatial and thermal responses generates nine potential spatial‐thermal patterns that can arise in response to the increase in temperatures. A species could temporarily shift its regional or local distribution to inhabit cooler locations and, at the same time, be observed at increasingly higher temperatures than the average of the assemblage (SA/TT pattern). This potential case would imply that the species is able to minimize the negative effects of climate change through its thermal plasticity, as well as to spatially modify its distribution in a more effective way than the average of the species of the assemblage. Species that follow this pattern will be the least likely to suffer negative consequences from a change in temperature. Spatial adaptation may occur without evidence of thermal tolerance or adjustment (SA/TC), so that a species would be observed following the overall thermal trend. In this situation, the change in geographical distribution would be the primary factor responsible for maintaining the temperature at which the species is observed. If spatial adaptation occurs at the same time as thermal adjustment (SA/TA), the species would progressively tend to inhabit colder localities but also colder conditions, as if the spatial shift strategy were insufficient. The absence of spatial adaptation may occur as a consequence of, for example, dispersal limitations, morphological/physiological impediments or biotic interactions. In these cases, species may withstand temperature increases (SC/TT), show no apparent effect (SC/TC), or physiologically or seasonally adjust their thermal requirements to the new conditions (SC/TA). If the species can show a discrepant spatial response but tolerates the new temperature conditions (SD/TT), we would be dealing with organisms capable of withstanding such temperature changes, even if they are compelled to inhabit warmer locations. If the probability of occurrence of a species temporarily increases in warmer locations but does not show a significant change in its temperature conditions (SD/TC), it is plausible to again hypothesize that we are dealing with species that are able to tolerate these temperature changes. Finally, an increase in the probability of occurrence in warmer localities together with an adjustment to inhabit cooler conditions (SD/TA) seems an unlikely scenario unless it involves a change in seasonal occurrence or the choice of microclimatic conditions capable of mitigating the increase in temperatures.

In a third step, it is necessary to compare the spatial (latitude, longitude, or elevation) and temperature information coming from the occurrences of each species with that of OT (Box 1). Although spatial and temperature data can be intertwined they are not necessarily interdependent. There can be spatial displacement of observations without a corresponding change in the temperature of those observations, and species may migrate while still tolerating different temperature ranges. Similarly, the temperature of observations may remain unchanged despite climate change, without any noticeable shifts in latitude or longitude by the effect of microhabitats or climatic regions within the same spatial area. As a result, the spatial and thermal components can offer complementary insights, suggesting the presence of temporal modifications in spatial distributions and shifts in the temperatures at which species are observed. In the case of spatial variables, the contrast between the data derived from the occurrences of a species and OT data would allow to identify whether the temporal occurrences reflect a process of spatial adaptation (SA), spatial discordance (SD) or spatial conformance (SC). SA is the expected and recognized “range shift” strategy of organisms in response to climate change (Parmesan, 2006). The probability of occurrence may temporally decrease at locations with “a priori” cooler temperatures if spatial discordance appears. Thus, when SD occurs, it is to be expected that other factors are affecting species distributions, independent of climate change. Finally, SC implies that the probability of occurrence follows the overall trend experienced by the complete dataset. Species that show SC are also candidates for having no conspicuous modifications in their ranges and for not being spatially affected by climate change. Nevertheless, species showing SC patterns would follow the same biases of the whole dataset and one would not be able to infer a particular response to climate change. Analogous to the spatial response, the thermal response of the whole dataset can be estimated. Each occurrence of a species has an associated temperature that corresponds to the locality and date of the occurrence. Once the corresponding overall trend in temperature was calculated, we again are able to estimate if the temperatures of the occurrences suggest thermal tolerance (TT), thermal adjustment (TA) or thermal conformance (TC) (Box 1). TT implies that the probability of occurrence increases temporally and exceeds the average value at warmer temperatures because the species likely tolerates the temperature changes experienced thus far. Under thermal adjustment, there is a negative relationship between temperature and date of occurrence, showing that species tend to temporarily inhabit progressively colder conditions. Both TT and TA responses may occur as a consequence of microevolutionary changes or phenotypic plasticity in the thermal response of a species (Whitman & Ananthakrishnan, 2009). As in the case of spatial variables, thermal conformance implies that the probability of occurrence does not vary temporally from the overall baseline trend of the full dataset. The species following this pattern are candidates for not being affected by changes in the temperature recorded thus far.

## 3 EXPECTATIONS IN THE CASE OF THE IBERIAN PENINSULA

**FIGURE 1:**
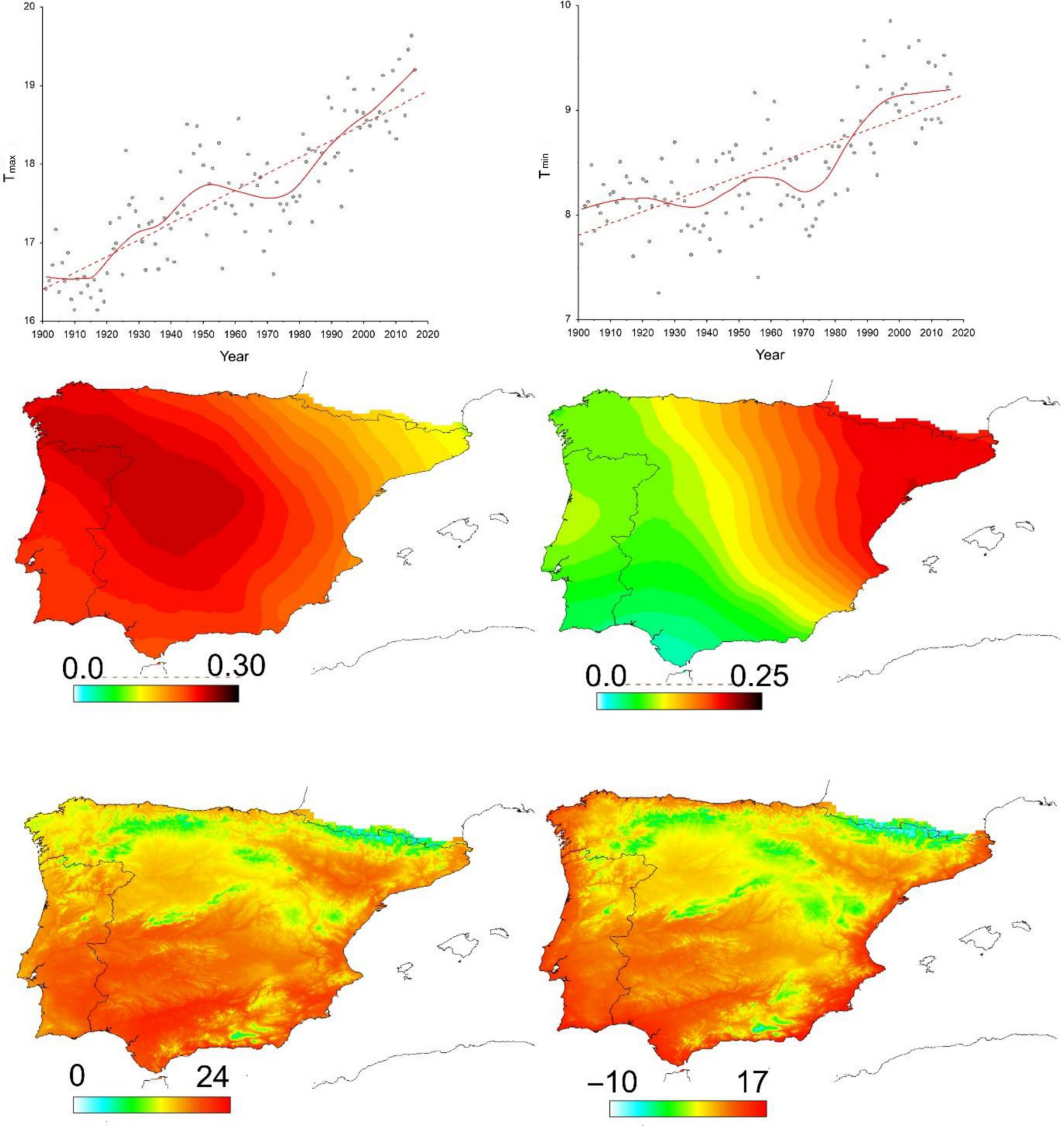
Annual variation in the mean maximum temperature (T_max_) and mean minimum temperature (T_min_) of all the 10 × 10 km UTM cells covering Iberia according to the CHELSA database (Karger et al., 2017). The dashed red lines represent the linear adjustment of the data, while the continuous line is the locally weighted smoothing. The maps represent the spatial patterns of the increase in the maximum (left) and minimum (right) temperatures from 1900 to 2016 (°C per decade) and the mean maximum and mean minimum temperature values during the study period (maps below).

The probability of occurrence may increase temporally at the highest elevations or northern most locations in the northern hemisphere, where temperatures are generally cooler. In the case of the Iberian Peninsula, the northern half of the region and mountainous areas have the coldest average temperatures (Chazarra‐Bernabé et al., 2020) (Figure 1). Thus, mean minimum and maximum temperatures for the period 1900–2016 decreased by 0.33 and 0.44°C by latitudinal degree, respectively. In the case of longitude, the geographical pattern of temperatures is not so obvious (Figure 1), but the mean minimum and maximum temperatures decreased 0.34°C and 0.20°C by longitudinal degree, being smaller in the east as a consequence of the longitudinally oriented eastern mountain ranges (Figure 1). Therefore, in the case of the Iberian Peninsula, we would expect an increase over time in the probability of occurrence in the north and east and at higher elevations, which would be reflected in a positive relationship significantly different from that of the overall trend between the date of occurrences and latitude, longitude or elevation. Furthermore, our approach assumes that an increase in temperature occurs in the target region along the temporal sequence at which occurrence data are collected. The CHELSA database (Karger et al., 2017) show that, in fact, positive increases of 0.21°C per decade in T_max_ and 0.11°C per decade in T_min_ occurred in the Iberian Peninsula during the period from 1900 to 2016 (Figure 1). Since fewer than 8% of all the considered database records were collected before 1970, we also estimated these temperature increases for the period 1970–2016 and found a 0.41°C increase per decade for T_max_ and a 0.28°C per decade increase for T_min_ (Figure 1).

## 4 METHODS

### 4.1 Data origin

We extracted data from an exhaustive database of Iberian dung beetles (see Lobo & Martín Piera, 1991; Sánchez‐Fernández et al., 2022), compiling species occurrences from the literature, museums, private collections, PhD theses, unpublished field records, and gray literature. The dung beetle species considered in this study belonged to the superfamily Scarabaeoidea, more specifically to the subfamilies Geotrupinae, Scarabaeinae, and Aphodiinae. These species primarily feed on mammal feces, although they may consume other types of food at various stages of their development (Krell & Moon, 2019). All the database records without a distinct locality or lacked information on the month and year of collection were discarded. All these incidence records were georeferenced at a 5‐min cell resolution (≈10 km × 10 km) using the web platform Google Maps when necessary. In total, 44,688 database records belonging to 215 species were compiled, selecting for subsequent analyses the 100 species with more than 100 records (41,521 database records; see Appendix 1). The year of sampling within the dataset ranges from 1901 to 2014.

CHELSA database (Karger et al., 2017) was used to download monthly mean temperature data at 0.5‐min resolution (≈1 km × 1 km) and mean values for each 5‐min cell were calculated. Thus, moth, year and geographic coordinates of each species database record is matched with the data of CHELSA in order to obtain two temperature values for each record: mean maximum temperature of the warmest month (T_max_) and the mean minimum temperature of the coldest month (T_min_). Thus, we obtain for each species the mean maximum and minimum temperature values of the locality of collection for the month and year in which it was observed. The elevation (Elev) of each database record at a resolution of 0.0423° (≈5 × 5 km) was determined from a digital elevation model downloaded from the USGS EROS Data Center (https://www.usgs.gov/centers/eros). Thus, two temperature variables (T_max_ and T_min_) reflecting the conditions at the time of collection, and three spatial variables (latitude or Lat, longitude or Long and elevation or Elev) were obtained for each of the database records.

### 4.2 Data treatment

We first estimated the slope of the linear relationships between the date of each record (the explanatory variable) and each one of the five temperature, location and elevation variables (the dependent variables) for the complete set of data to obtain the overall trend. To do that, monthly and yearly values were converted to a single decimal value (e.g., January and December of 1970 are 1970.0 and 1970.9, respectively). These general slopes are significantly different from zero for Lat (−0.07° per decade; *t* = −11.77; *p* < .0001), Long (−0.02° per decade; *t* = −3.16; *p* = .001), Elev (49.9 m per decade; *t* = 35.01; *p* < .0001) and T_min_ (−0.23°C per decade; *t* = −16.54; *p* < .0001) but not for T_max_ (−0.004°C per decade; *t* = −0.23; *p* = .82). Thus, these overall trends indicate that the probability of obtaining data on Iberian dung beetles has changed over time, being greater at higher elevation localities but also at southern localities. The negative relationship observed for longitude suggests a temporal shift in the frequency of collections towards the western part of Iberia. The minimum mean temperatures of the complete set of occurrences tended to decrease over time, but the mean maximum temperatures remained unchanged during the considered period.

Once these overall trends were calculated, slopes linking these five dependent variables to the date of each record were calculated individually for each dung beetle species. A homogeneity of slopes model directed to examine whether the continuous and categorical predictors interact in influencing responses (Quinn & Keough, 2002) was then used to test whether the overall and individual slopes differ. We used an exigent associated probability (*p* ≤ .01) to determine if the temporal response of the data of a specific species differed from those of the overall trend. As we study 100 species, we anticipate the possibility of one species being a false positive, which could lead to a statistically significant difference between the slopes, erroneously suggesting that this species was affected by temperature changes. Nonetheless, the *p*‐values are regarded here as “clarity evidences” (Dushoff et al., 2019), enabling us to infer that those species displaying “statistically significant differences” are strong candidates for experiencing temporal variations in their frequency of occurrence across spatial and thermal gradients. The obtained results allowed the identification of the spatiotemporal pattern followed by each species. In the case of spatial variables, the slope with the highest absolute standardized value was selected when there were two or more slopes that were statistically significant.

All the considered species were grouped according to their taxonomic rank as Aphodiinae (55 species), Scarabaeinae (34 species), and Geotrupidae (11 species), and according to their feeding behavior (Tonelli, 2021; Zunino & Palestrini, 1986). Scarabaeinae and Geotrupidae species mostly dig vertical tunnels in the ground in which they bury dung (hypophagic behavior or tunnelers). Aphodiinae species are smaller in size and live and feed inside excrement (endophagic behavior or dwellers). Due to the tunneler behavior of *Colobopterus erraticus* and *Teuchestes fossor* on some occasions, these two Aphodiinae species were also considered hypophagic (Rojewski, 1983; Zunino & Barbero, 1990). Last, telephagic Scarabaeinae and Geotrupidae species (Zunino & Palestrini, 1986) make brood masses or balls that are transported, buried or deposited at some distance from the dung source (commonly known as rollers). The fresh biomass of each species was estimated using the length‐to‐body weight relationship for dung beetles (Lobo, 1993; *r* = .989, *p* < .001). Body length was obtained from the literature (Baraud, 1977) and original descriptions of the species. Finally, the temporal occurrence trend of each species was estimated by computing the relative frequency of the database records of each species in each year since 1950 and calculating the slope of these frequencies against the year. The frequency distribution of the different spatio‐thermal patterns for the different taxonomic and trophic qualitative categories was analyzed by a chi square test of homogeneity. The differences in body weight and number of database records between spatio‐thermal patterns were assessed using the Kruskal–Wallis nonparametric rank test. The purpose of these analyses was to examine whether the prevalence of the different spatio‐thermal patterns is associated with life history/ecological differences between feeding guilds, and body weight.

## 5 RESULTS

Twenty species (20% of the total) do not have any temporal trends that differed from those displayed by the whole dataset. Twenty‐eight other species do not show a difference in thermal response relative to the overall trend and have a discordant spatial response (significant negative slope or SD; Table 1; see Appendix 1). The relative frequencies of the three subfamilies for these 48 species do not differ from the frequencies of the complete set of data (*χ* ^2^ value = 2.75; df = 2; *p* = .25). However, the frequencies of the three trophic groups do differ (*χ* ^2^ value = 7.97; df = 2; *p* = .02) because seven out of the eight telephagic species appear in these categories (Table 1).

**TABLE 1:** Species richness (S), mean number of database records (R; ± 95% CI), mean body weight (in mg; BW) and mean occurrence temporal trend (OT; percentage of the total database records per decade) of the species following each of the nine spatial‐thermal patterns (see Box 1).

		Spatial		
		Adaptation (SA)	Conformance (SC)	Discordance (SD)
Thermal	Tolerance (TT)	7	5	10
	Conformance (TC)	26	20	28
	Adjustment (TA)	4	0	0

	SA and TT	SA and TC	SA and TA	SC and TT	SD and TT	SC and TC	SC and TA	SD and TC	SD and TC	
S	7	26	4	5	10	20	0	28	0	
R	701 ± 255	480 ± 132	564 ± 337	647 ± 301	347 ± 213	344 ± 151	0	295 ± 127	0	KW = 21.21; *p* = .002
BW	11.8 ± 116.1	22.2 ± 60.3	52.5 ± 153.6	8.7 ± 137.4	54.9 ± 97.2	108.2 ± 68.7	0	98.7 ± 58.1		KW = 5.90; *p* = .43
OT	0.12 ± 0.19	−0.02 ± 0.10	−0.11 ± 0.25	0.11 ± 0.22	−0.05 ± 0.16	−0.08 ± 0.11	0	−0.03 ± 0.09	0	KW = 11.47; *p* = .08
A	7	17	1	4	5	10	0	11	0	*χ* ^2^ = 43.34; *p* < .001
S	0	7	3	1	3	7	0	13	0	*χ* ^2^ = 41.71; *p* < .001
G	0	2	0	0	2	3	0	4	0	*χ* ^2^ = 16.00; *p* = .04
E	6	17	1	3	5	10	0	11	0	*χ* ^2^ = 45.57; *p* <.001
H	1	8	3	2	5	7	0	13	0	*χ* ^2^ = 35.35; *p* < .001
T	0	1	0	0	0	3	0	4	0	*χ* ^2^ = 21.25; *p* = .007

*Note*: The colors aim to illustrate the locations of the patterns within the upper contingency table, where blank cells represent patterns that do not show a species response to temporal changes in temperature. The number of Aphodiinae (A), Scarabaeinae (S), Geotrupidae (G), endophagic (E), hypophagic (H) and telephagic (T) species are also included, as well as the results of Kruskal–Wallis (KW) nonparametric rank tests and chi square tests of homogeneity between taxonomic and trophic qualitative categories.

**FIGURE 2:**
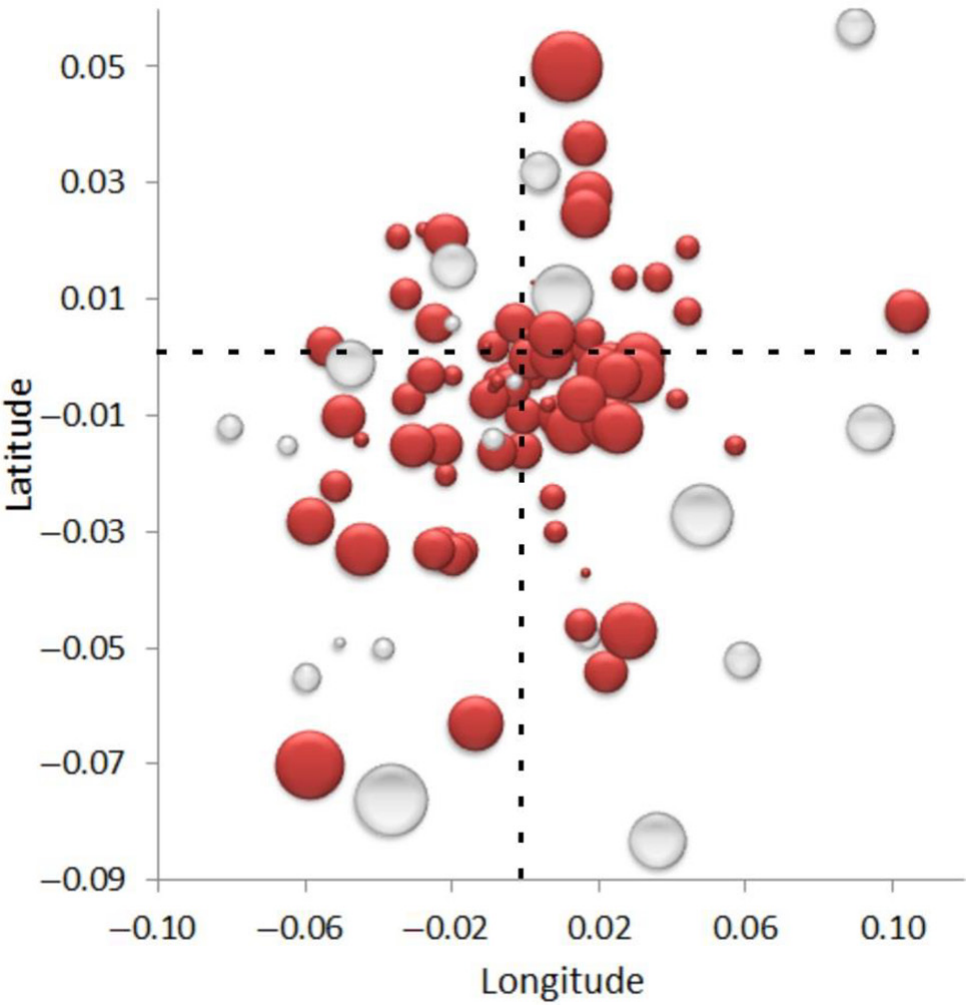
Relationships of the slopes of the linear regressions between the latitude, longitude and elevation of dung beetle occurrences and the date they were collected. The size of the dots represents the value of the slope describing the relationship with elevation (red dots = positive slopes; white dots = negative slopes). The dashed lines indicate the places at which no temporal latitudinal and longitudinal changes occurred.

**FIGURE 3:**
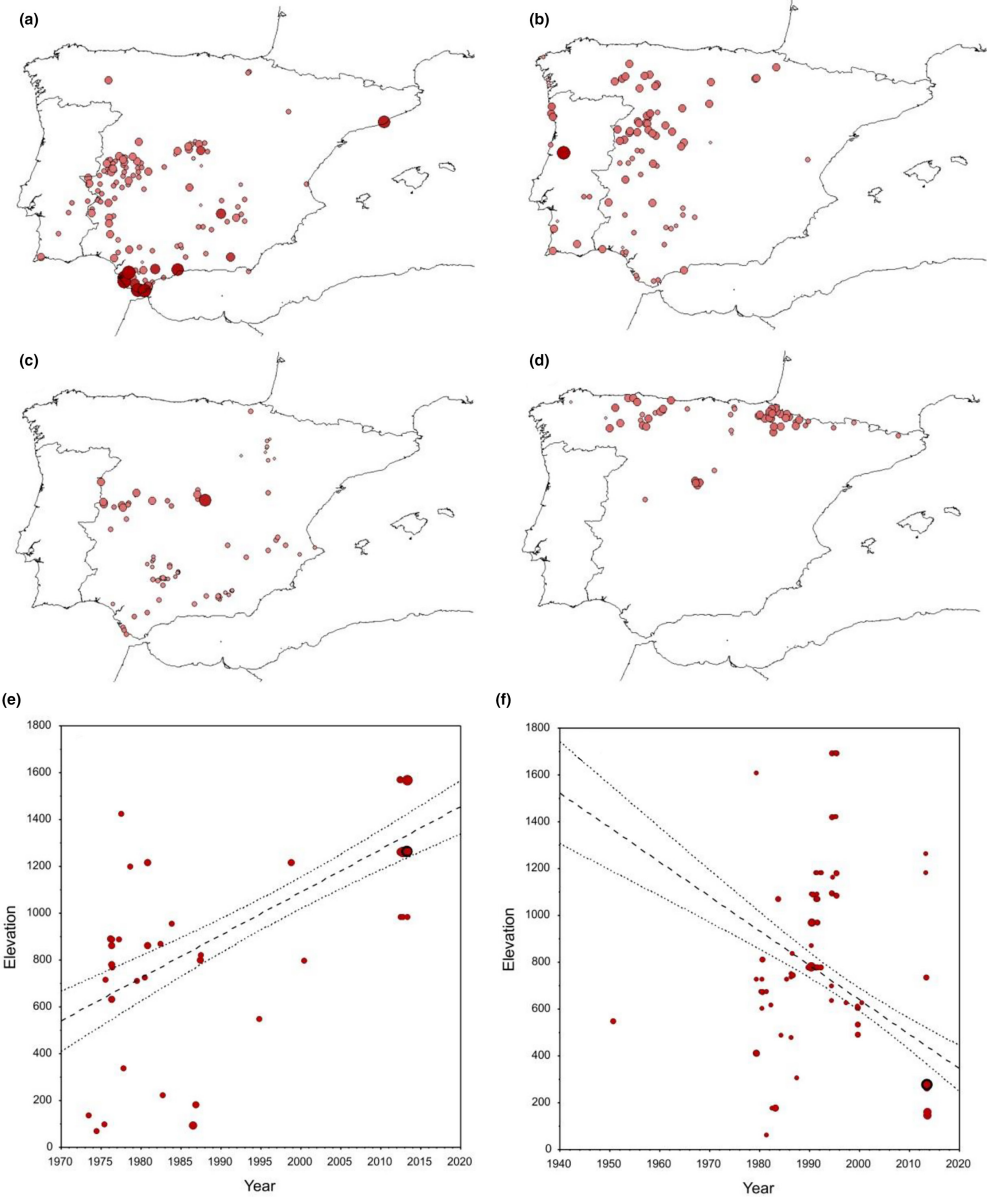
Iberian distribution of a dung beetle species with positive (a; *Bubas bison*) and negative (b; *Ceratophyus hoffmannseggi*) relationships between latitude and collection date, and with positive (c; *Euorodalus tersus*) and negative (d; *Anoplotrupes stercorosus*) relationships between longitude and collection date. The larger dots represent older occurrences. Figure (e, f) shows the relationship between collection date (Year) and elevation for a dung beetle species with a positive relationship (*Onthophagus coenobita*) and another with a negative relationship between these two variables (*O. merdarius*). The size of the dots represents the number of occurrences with the same values.

Three out of each four species studied (*n* = 75) have a linear trend in any one of the three considered spatial variables that differs significantly (*p* ≤ .01) from those of the complete dataset (Appendix 1). However, almost half of these species show a negative trend (*n* = 38; spatial discordance or SD), and the other half show a positive trend (*n* = 37; spatial adaptation or SA). The spatial variable along which the highest number of species vary temporally is longitude (*n* = 40), followed by elevation (*n* = 36) and latitude (*n* = 29) (Figure 2). However, 59%, 50% and 42% of the statistically significant temporal variations along the studied latitude, longitude and elevation are negative, respectively (Figure 3). Only two species (*Onthophagus similis* and *Melinopterus consputus*) had significantly temporally modified distributions along the three considered spatial gradients at the same time. Most of the species showed significant spatial variation over time in only one of the three spatial variables.

Twenty‐six species (26% of total) show a statistically significant temporal variation in the temperature values of their occurrences: five exclusively in the case of maximum temperature, six exclusively for minimum temperature, and 15 for both temperature variables at the same time (Table 1; Appendix 1). Most of these species (*n* = 22; 85%) show a statistically significant and positive increase in their temperature values over time (thermal tolerance or TT). Of these 22 species, 86% (*n* = 19) have a positive trend in their temporal occurrence (Appendix 1). Two species have a temporal decrease in the maximum temperature of their occurrences (*O. similis* and *O. stylocerus*), and two species experience a significant decrease in the minimum temperatures of their occurrences (*Labarrus lividus* and *Bubas bison*). Of these four TA (thermal adjustment) species, three show a negative trend in their temporal occurrence (Appendix 1).

The distribution of species between the nine spatial‐thermal patterns is not homogenous (*χ* ^2^ = 84.6; df = 8; *p* < .0001). Most of the studied species do not show temperature slopes with temporal trends that differ significantly from the overall trends (thermal conformance or TC; 74%), whether the temporal trends of the spatial variables are positive (spatial adaptation; 26%), negative (spatial discordance; 28%) or similar to the overall trend (spatial conformance; 20%) (Table 1). A positive temporal increase in the temperature of dung beetle occurrences is more frequent (thermal tolerance; 22%) than a negative trend (thermal adjustment; 4%). Most species appear to be associated with thermal adjustment and spatial conformance or spatial discordance conditions, and very few appear to be associated with thermal tolerance/spatial adaptation, thermal tolerance/spatial conformance, or thermal adjustment/spatial adaptation (Table 1). The distributions of Aphodiinae and Scarabaeinae species between the nine spatial‐thermal patterns are also not homogenous, nor are the distributions of endophagic, hypophagic or telephagic species (Table 1) due mainly to the high proportion of endophagic Aphodiinae with spatial adaptation and the high proportion of hypophagic and Scarabaeinae with spatial discordance patterns. The temporal trend values of species occurrence and the mean body weight of the species do not differ between the spatial‐thermal patterns, but the average number of database records differs (Table 1).

## 6 DISCUSSION

In this study, we propose a method to utilize occurrence data already available in databases for any group of organism and large‐scale interpolated temperature values derived from climate station data. By using these two sources of information, we aim to estimate whether existing records of Iberian dung beetle species exhibit a spatial or thermal pattern that significantly deviates from the overall trend. We propose that this enables us to infer specific responses to temperature changes. The proposal seeks to leverage the vast amount of opportunistic data collected over decades of uncoordinated collection efforts to examine potential species responses to one of the most pressing environmental challenges currently facing humanity and ecosystems. While not definitive evidence, the results generated by this approach should be regarded as preliminary information that can be used to formulate hypotheses about different species' responses to climate change.

Two primary factors have shaped the outcomes of our study: the identified temperature trend and the observed spatial and thermal tendencies within the complete dataset (i.e., overall trends). These factors are reliant not only on the specific species assemblage under consideration but also on the chosen region and the time period studied, which are in turn influenced by the characteristics of the compiled and available data. When occurrence data cover a substantial region and/or a lengthy time span, it is more probable for significant patterns to emerge, owing to the broader representation of the climatic gradient encompassed by the data. Prior research has leveraged comparisons between individual trends and overall trends to detect meaningful patterns (Telfer et al., 2002), a critical approach that underpins our methodology. In our study, we did not interpret the overall trends in isolation as definitive patterns; rather, we utilized them as reference points to infer the potential significance of each individual pattern. This comparison forms a crucial element of our proposed methodology. As previously highlighted, overall trends can be influenced by inherent biological and environmental factors, as well as potential temporal biases in data collection. In both scenarios, comparing individual trends with the overall trend lends credibility to the results and strengthens the inferences drawn.

Let us consider a hypothetical scenario where data for each year come from a well‐designed and standardized survey. In this case, the temporal variations in spatial and thermal data would constitute an unbiased sample reflecting temperature fluctuations in species occurrences. If the complete dataset follows the expected trend in response to a temperature changes (e.g., a latitudinal shift), species exhibiting a noticeable and more pronounced linear slope in the relationship between year and latitude would be considered to undergo spatial adaptation. Consequently, only species displaying a distinct pattern of change would be deemed to undergo a latitudinal shift in response to temperature fluctuations, even when the overall trend suggest that spatial adaptation is the predominant response for most species. Now, let us contemplate a situation where data are collected through temporally biased surveys. If recent sampling efforts have primarily focused on the northern part of a region situated in the Northern Hemisphere, the biased effect would mimic the impact of climate change on organisms. In such cases, only those effects that deviate from the overall trend would be considered significant for each species. Therefore, only those species showing a clear tendency to inhabit more northern locations over time would be deemed affected by climate change. Conversely, if recent survey endeavors have exhibited a bias towards the south, current species data would demonstrate a contrary direction to the expected impact of climate change. In such instances, species data would need to distinctly counter the general dataset pattern. Hence, the implications derived from contrasting the overall trend of all the data with the individual trend of each species are stringent, suggesting that a species has undergone a temporary shift in its spatial distribution or thermal niche. In practice, this comparison of slopes would likely yield false negatives (i.e., data indicating non‐significant differences in slopes from species that have undergone temporal changes). Thus, comparing individual and overall trends requires the variation pattern to be notably robust to substantiate the hypothesis that a species is undergoing spatial or thermal changes in response to temperature fluctuations, thereby minimizing the potential impact of biases on the dataset.

In the case of Iberian dung beetles, previous studies have indicated that the opportunistically collected occurrence data are clearly biased both spatially and climatically (Hortal et al., 2008; Lobo et al., 2007; Sánchez‐Fernández et al., 2022). The oldest observations of dung beetles are mainly restricted to the north‐eastern Iberian corner of the Iberian Peninsula (Lobo et al., 2007), while recent observations are more likely in the south and west of the Iberian Peninsula. Previous studies have also shown that temperatures have undergone increases that are not homogeneous across the Iberian territory (Lorenzo et al., 2021; Peña‐Angulo et al., 2021). The mean maximum temperature increased more in the central region and in the northwestern Atlantic region (Figure 1), a pattern that coincides with the temporal increase in heatwave intensity (Lorenzo et al., 2021). However, mean minimum temperatures increased more in the eastern Mediterranean region (Figure 1; see also Peña‐Angulo et al., 2021). Under this scenario of biased survey effort and a temporary increase in temperatures, our results indicate that approximately half of the species would have experienced a response to these temporal changes in temperature. Among the species that do not show any response to temperature variation, those having hypophagic and telephagic trophic behaviors dominate (56%), while endophagic species more frequently show a temporal temperature response (65%). The detected response patterns can be explained by considering the behavioral and ecological characteristics of the species. Thus, our results show that the spatial response is the most frequent response; seven out of 10 species that reacted to temperature variations did so spatially (spatial adaptation). Aphodiinae species such as *Chilothorax distinctus*, *Labarrus lividus*, *Esymus pusillus,* and *Nobiellus bonnairei* or Scarabaeinae species such as *Euonthophagus gibbosus* are among those with the greatest changes in spatial and elevational distributions in response to temperature variations. Of the 37 species showing spatial adaptation, 25 are Aphodiinae (68%), suggesting that not burying the dung and not constructing brood nests underground protected from environmental conditions (i.e., a free lifestyle) could favor a spatial escape strategy in overcoming changes in temperature. Thus, the greater sensitivity of Aphodiinae species to environmental conditions favors a spatial response to changes in temperature. This greater sensitivity to environmental changes among Aphodiinae species would also be manifested in their ability to modify their phenology (Cuesta et al., 2021) or the alteration of their populations in response to a decrease in trophic resources (Tonelli et al., 2019). According to our results, the most frequent spatial adaptation strategy is related to an increase in the elevation of the occurrences, although changes due to differences in both latitude and longitude are also frequent. This result is in agreement with the steeper gradient of temperature that implies a change in elevation compared to a change in latitude (Colwell et al., 2008). Other studies recorded an uphill retreat of montane dung beetle species (Birkett et al., 2018; Menéndez et al., 2014) and other Iberian insects (Wilson et al., 2005, 2007). Evidence of latitudinal or longitudinal Iberian shifts is elusive (Mingarro et al., 2021), although it has been found in other territories with much more complete data series and a long history of biological studies (Hickling et al., 2006). According to these results (Appendix 1), it is possible to select some candidate species for which the study of the ecology and genetics of the populations at the distribution margins may help to elucidate their recent expansion (Parmesan, 2006).

The thermal temporal response of Iberian dung beetle species appears in 26 species, but only in 15 (58%) is this response present without the joint participation of spatial adaptation responses. The thermal adjustment of a species to cooler temperature conditions without experiencing spatial adaptation seems to be a rare strategy. Thus, the ability of a species to live under colder conditions without spatial modifications of the distribution may require physiological, phenological or diel changes that are difficult to produce. Mediterranean dung beetle species can only partially vary their phenological occurrence to mitigate the effects of climate change (Cuesta et al., 2021). Furthermore, the thermal responses of dung beetle species to different niche dimensions are often unrelated (Calatayud et al., 2021), so fulfilling the temperature requirements for one niche dimension may result in negative effects on other dimensions. The low relevance of thermal adjustment patterns are thus evidence of the preeminence of spatial responses to climate change and the importance of maintaining a matrix of natural habitats that allows organisms to disperse. Iberian dung beetle species seem much more likely to tolerate ongoing increases in temperature. Interestingly, most (86%) of the species that showed a thermal tolerance pattern also showed a positive temporal occurrence trend. Those species that were not influenced by temperature increases showed a higher frequency of occurrence over time. Among these species are *Ceratophyus hoffmannseggi*, *Onthophagus merdarius*, *Rhodaphodius foetens* or *Onthophagus latigena*, all of which can withstand temperature changes so well that they would have increased their chances of collection.

In this proposal, we have selected candidate species capable of overcoming the challenges posed by changes in temperatures observed thus far. These candidate species may be the focus of future studies that delve into this topic more specifically and comprehensively. However, our proposal enables us to identify only a subset of the species experiencing population declines. Clearly, a low proportion of species exhibiting spatial adaptation and/or thermal tolerance suggests that the impacts of climate change are particularly severe. In the case of Iberian dung beetles, the results indicate that roughly half of the species may be negatively affected by rising temperatures. Importantly, the proposed approach can be applied to many other taxonomic groups and environmental variables, allowing for global and comparative assessments of how these taxa will cope with different variables representing climate change.

## AUTHOR CONTRIBUTIONS

**Jorge M. Lobo:** Conceptualization (lead); formal analysis (lead); writing – original draft (lead). **Mario Mingarro:** Formal analysis (equal); writing – review and editing (equal). **Martin Godefroid:** Writing – review and editing (equal). **Emilio García‐Roselló:** Formal analysis (equal); writing – review and editing (equal).

## CONFLICT OF INTEREST STATEMENT

The authors declare that they have no known competing financial interests or personal relationships that could have appeared to influence the work reported in this paper.

## Data Availability

All the used data are freely available in http://geobrink.uclm.es/Geobrink/.

## APPENDIX 1

### 1.1

Main data of the 100 Iberian dung beetle species. R = number of database records. F = family/subfamily (A = Aphodiinae, S = Scarabaeinae, G = Geotrupidae), T = trophic group (E = endophagic, H = hypophagic, T = telephagic). BW = body weight (in mg). OT = occurrence temporal trend (in % of total database records per decade). The slopes of the linear relationships between the date of the collections and latitude (Lat), longitude (Long), elevation (Elev), mean maximum temperature of the warmest month (T_max_) and mean minimum temperature of the coldest month (T_min_) of species database records are included, indicating with “Y” when these slopes are significantly different (*p* ≤ .01) for the ones representing the complete set of analyzed data. The existence of significant positive (+), significant negative (−) or non‐significant relationships (=) is outlined for spatial (S) and thermal (T) variables, as well as the spatial‐thermal pattern at which each species is ascribed (see Box 1).

**Table:**

Species	R	F	TG	BW	CT	Latitude	Longitude	Elevation	T_max_	T_min_	S	T	Pattern
*Agrilinus ibericus* (Harold, 1874)	334	A	E	1.6	0.04	−0.046	0.015	3.886	−0.081	−0.060	=	=	Spatial and thermal conformance
*Anomius castaneus* (Illiger, 1803)	257	A	E	6.1	−0.13	−0.007	−0.032	3.947	0.000	−0.083	=	=	Spatial and thermal conformance
*Aphodius fimetarius* (Linnaeus, 1758)	2207	A	E	5.4	−0.86	−0.010	−0.001	5.393	0.015	−0.015	=	=	Spatial and thermal conformance
*Bodilus longispina* (Küster, 1854)	128	A	E	8.7	−0.22	−0.032	−0.023	4.481	−0.037	−0.064	=	=	Spatial and thermal conformance
*Bodilus lugens* (Creutzer, 1799)	182	A	E	13.1	−0.18	0.006	−0.003	6.407	−0.018	−0.029	=	=	Spatial and thermal conformance
*Esymus merdarius* (Fabricius, 1775)	374	A	E	1.6	−0.03	0.009	0.010	5.542	−0.030	−0.041	=	=	Spatial and thermal conformance
*Eudolus quadriguttatus* (Herbst, 1783)	231	A	E	1.6	−0.10	−0.005	−0.004	5.897	0.005	−0.026	=	=	Spatial and thermal conformance
*Heptaulacus testudinarius* (Fabricius, 1775)	111	A	E	0.7	0.01	−0.037	0.016	0.362	0.071	0.051	=	=	Spatial and thermal conformance
*Phalacronothus quadrimaculatus* (Linnaeus, 1761)	183	A	E	0.7	0.02	−0.030	0.008	1.869	−0.032	−0.062	=	=	Spatial and thermal conformance
*Subrinus vitellinus* (Klug, 1845)	127	A	E	0.5	0.00	−0.012	0.094	−8.571	−0.132	0.099	=	=	Spatial and thermal conformance
*Chelotrupes momus* (Olivier, 1789)	203	G	H	96.2	0.22	0.021	−0.035	2.434	0.033	0.076	=	=	Spatial and thermal conformance
*Geotrupes mutator* (Marsham 1802)	453	G	H	224.0	0.70	−0.010	0.009	6.660	−0.026	−0.037	=	=	Spatial and thermal conformance
*Thorectes lusitanicus* (Jekel, 1866)	121	G	T	101.4	0.23	−0.008	0.018	4.814	−0.063	−0.069	=	=	Spatial and thermal conformance
*Bubas bubalus* (Olivier, 1811)	564	S	H	173.0	−0.25	−0.003	0.002	2.947	−0.001	−0.028	=	=	Spatial and thermal conformance
*Cheironitis ungaricus* (Herbst, 1789)	142	S	H	171.4	−0.05	0.003	0.002	3.238	0.008	−0.009	=	=	Spatial and thermal conformance
*Copris lunaris* (Linnaeus, 1758)	560	S	H	143.8	−0.34	−0.016	−0.008	5.899	−0.021	−0.047	=	=	Spatial and thermal conformance
*Onitis ion* (Olivier, 1789)	199	S	H	76.2	−0.15	−0.010	0.011	2.895	−0.003	−0.028	=	=	Spatial and thermal conformance
*Onthophagus grossepunctatus* Reitter, 1905	168	S	H	2.3	−0.14	−0.012	0.012	9.908	0.033	−0.007	=	=	Spatial and thermal conformance
*Scarabaeus sacer* Linnaeus, 1758	175	S	T	1120.5	0.00	−0.004	−0.004	5.403	−0.007	−0.043	=	=	Spatial and thermal conformance
*Sisyphus schaefferi* (Linnaeus, 1758)	168	S	T	15.9	−0.39	0.002	−0.009	3.711	0.016	−0.007	=	=	Spatial and thermal conformance
*Acanthobodilus immundus* (Creutzer, 1799)	270	A	E	3.1	0.03	−0.010	0.026	−2.707 Y	−0.023	−0.033	−	=	Spatial discordance and thermal conformance
*Acrossus depressus* (Kugelann, 1792)	446	A	E	10.7	0.21	−0.020	−0.022 Y	1.959	0.022	0.004	−	=	Spatial discordance and thermal conformance
*Acrossus luridus* (Fabricius, 1775)	678	A	E	10.7	−0.02	−0.024 Y	0.007	2.430	0.026	−0.008	−	=	Spatial discordance and thermal conformance
*Alocoderus hydrochaeris* (Fabricius, 1798)	121	A	E	10.7	−0.18	−0.055 Y	−0.060 Y	−2.975	−0.011	0.026	−	=	Spatial discordance and thermal conformance
*Ammoecius lusitanicus* Erichson, 1848	198	A	E	1.6	0.13	0.011	0.010	−14.082 Y	0.002	0.020	−	=	Spatial discordance and thermal conformance
*Calamosternus hyxos* (Petrovitz, 1962)	173	A	E	1.3	0.27	0.032	0.004	−5.932 Y	0.029	0.018	−	=	Spatial discordance and thermal conformance
*Melinopterus abeillei* (Sietti, 1903)	187	A	E	3.1	−0.04	0.016	−0.020	−7.991 Y	−0.041	−0.058	−	=	Spatial discordance and thermal conformance
*Melinopterus prodromus* (Brahm, 1790)	298	A	E	3.5	−0.16	0.021 Y	−0.022 Y	7.414	−0.001	−0.031	−	=	Spatial discordance and thermal conformance
*Nialus varians* (Duftschmid, 1805)	105	A	E	3.6	−0.26	0.001	−0.053 Y	2.504	−0.005	−0.022	−	=	Spatial discordance and thermal conformance
*Subrinus sturmi* (Harold, 1870)	217	A	E	0.7	0.03	−0.083 Y	0.036	−12.660 Y	−0.049	−0.022	−	=	Spatial discordance and thermal conformance
*Volinus sticticus* (Panzer, 1798)	210	A	E	1.6	0.15	−0.034 Y	−0.020	6.211	0.007	−0.028	−	=	Spatial discordance and thermal conformance
*Anoplotrupes stercorosus* (Scriba, 1790)	154	G	H	96.2	0.64	0.002	−0.055 Y	5.497	0.027	−0.015	−	=	Spatial discordance and thermal conformance
*Geotrupes stercorarius* (Linnaeus, 1758)	344	G	H	263.3	0.20	−0.015	−0.023 Y	6.456	−0.013	−0.035	−	=	Spatial discordance and thermal conformance
*Sericotrupes niger* (Marsham, 1802)	164	G	H	143.8	0.75	−0.050 Y	−0.039	−1.768	0.001	0.013	−	=	Spatial discordance and thermal conformance
*Typhaeus typhoeus* (Linnaeus, 1758)	437	G	H	157.9	0.15	−0.054 Y	0.022 Y	7.126	−0.020	−0.047	−	=	Spatial discordance and thermal conformance
*Ateuchetus laticollis* Linnaeus, 1767	164	S	T	176.1	−0.46	0.022 Y	−0.028 Y	0.926	−0.001	−0.019	−	=	Spatial discordance and thermal conformance
*Ateuchetus puncticollis* (Latreille, 1819)	119	S	T	243.1	−0.01	−0.033 Y	−0.025 Y	6.497	0.004	−0.032	−	=	Spatial discordance and thermal conformance
*Ateuchetus semipunctatus* Fabricius, 1792	102	S	T	587.5	−0.23	−0.049 Y	−0.051	−0.474 Y	0.005	0.017	−	=	Spatial discordance and thermal conformance
*Copris hispanus* (Linnaeus, 1764)	462	S	H	331.0	0.36	−0.003	−0.020 Y	1.634	0.012	−0.010	−	=	Spatial discordance and thermal conformance
*Euoniticellus pallipes* (Fabricius, 1798)	121	S	H	11.6	−0.02	−0.012	−0.081	−2.538 Y	0.030	0.007	−	=	Spatial discordance and thermal conformance
*Euonthophagus amyntas* (Olivier, 1789)	447	S	H	19.0	−0.55	0.011 Y	−0.033 Y	3.771	0.022	−0.010	−	=	Spatial discordance and thermal conformance
*Gymnopleurus flagellatus* (Fabricius, 1787)	280	S	T	50.3	−0.46	−0.003	−0.029 Y	2.688	0.020	−0.010	−	=	Spatial discordance and thermal conformance
*Onitis belial* Fabricius, 1798	285	S	H	602.2	−0.18	−0.014	−0.009	−1.831 Y	−0.038	−0.024	−	=	Spatial discordance and thermal conformance
*Onthophagus joannae* Goljan, 1953	220	S	H	2.3	0.00	−0.022	−0.052 Y	3.841	−0.028	−0.053	−	=	Spatial discordance and thermal conformance
*Onthophagus lemur* (Fabricius, 1781)	414	S	H	5.4	−0.14	−0.015	−0.031 Y	7.488	0.002	−0.029	−	=	Spatial discordance and thermal conformance
*Onthophagus maki* (Illiger, 1803)	158	S	H	5.8	−0.16	−0.015	−0.065	−1.521 Y	0.001	−0.022	−	=	Spatial discordance and thermal conformance
*Onthophagus ruficapillus* Brullé, 1832	299	S	H	2.5	−0.25	−0.033 Y	−0.018 Y	4.609	−0.015	−0.044	−	=	Spatial discordance and thermal conformance
*Onthophagus taurus* (Schreber, 1759)	1198	S	H	14.6	−0.72	0.006 Y	−0.025 Y	5.739	0.013	−0.019	−	=	Spatial discordance and thermal conformance
*Labarrus lividus* (Olivier, 1789)	219	A	E	1.3	0.07	0.050 Y	0.011	19.252 Y	−0.038	−0.124 Y	+	‐	Spatial adaptation and thermal adjustment
*Bubas bison* (Linnaeus, 1767)	370	S	H	173.0	−0.15	0.037 Y	0.016	7.399	−0.048	−0.075 Y	+	‐	Spatial adaptation and thermal adjustment
*Onthophagus similis* (Scriba, 1790)	1458	S	H	3.1	−0.17	0.004 Y	0.007 Y	8.678 Y	−0.023 Y	−0.043 Y	+	‐	Spatial adaptation and thermal adjustment
*Onthophagus stylocerus* Graëlls, 1851	209	S	H	32.7	−0.21	−0.007	0.015 Y	9.130 Y	−0.051 Y	−0.072 Y	+	‐	Spatial adaptation and thermal adjustment
*Acrossus carpetanus* (Graëlls, 1847)	207	A	E	68.6	−0.18	0.013 Y	0.002	0.102 Y	0.009	0.002	+	=	Spatial adaptation and thermal conformance
*Acrossus rufipes* (Linnaeus, 1758)	478	A	E	41.2	−0.09	−0.004	−0.008	0.838 Y	−0.003	0.002	+	=	Spatial adaptation and thermal conformance
*Amidorus obscurus* (Fabricius, 1792)	212	A	E	6.9	0.05	−0.005	−0.009	0.543 Y	0.030	0.008	+	=	Spatial adaptation and thermal conformance
*Ammoecius elevatus* (Olivier, 1789)	581	A	E	6.9	−0.12	0.004	0.000	0.465 Y	0.025	0.004	+	=	Spatial adaptation and thermal conformance
*Ammoecius frigidus* Brisout, 1866	320	A	E	1.6	−0.03	−0.012	0.025	9.585 Y	0.009	−0.020	+	=	Spatial adaptation and thermal conformance
*Anomius annamariae* (Baraud, 1982)	419	A	E	4.9	−0.12	−0.004	0.021 Y	1.844	0.007	−0.002	+	=	Spatial adaptation and thermal conformance
*Bodilus ictericus* (Laicharting, 1781)	699	A	E	1.6	0.10	0.008	0.044 Y	2.960	0.033	−0.001	+	=	Spatial adaptation and thermal conformance
*Calamosternus unicolor* (Olivier, 1789)	112	A	E	3.1	0.02	−0.007	0.041 Y	1.728	−0.018	−0.040	+	=	Spatial adaptation and thermal conformance
*Chilothorax distinctus* (Muller, 1776)	761	A	E	1.6	0.10	−0.047 Y	0.028	12.344 Y	0.011	−0.048	+	=	Spatial adaptation and thermal conformance
*Chilothorax lineolatus* (Illiger, 1803)	387	A	E	1.1	0.11	0.028 Y	0.017	8.671	0.036	−0.028	+	=	Spatial adaptation and thermal conformance
*Esymus pusillus* (Herbst, 1789)	187	A	E	1.1	0.17	−0.033 Y	−0.045	10.840 Y	0.049	0.004	+	=	Spatial adaptation and thermal conformance
*Euorodalus tersus* (Erichson, 1848)	283	A	E	1.1	0.11	0.008	0.104 Y	7.468	−0.024	−0.076	+	=	Spatial adaptation and thermal conformance
*Melinopterus sphacelatus* (Panzer, 1798)	1029	A	E	2.6	−0.04	−0.003	0.025	8.898 Y	−0.012	−0.038	+	=	Spatial adaptation and thermal conformance
*Nimbus contaminatus* (Herbst, 1783)	588	A	E	4.1	0.10	0.004	0.017 Y	4.206	−0.031	−0.036	+	=	Spatial adaptation and thermal conformance
*Nimbus proximus* Ádám, 1994	812	A	E	3.6	0.06	−0.001	0.023 Y	7.648	−0.025	−0.032	+	=	Spatial adaptation and thermal conformance
*Nobiellus bonnairei* (Reitter, 1892)	134	A	E	0.9	0.18	0.057	0.090 Y	−5.627	0.146	0.101	+	=	Spatial adaptation and thermal conformance
*Trichonotulus scrofa* (Fabricius, 1787)	335	A	E	0.7	0.05	−0.003	0.030	12.157 Y	0.014	−0.017	+	=	Spatial adaptation and thermal conformance
*Geotrupes ibericus* Baraud, 1958	680	G	H	263.3	−0.15	−0.002	0.020	8.370 Y	−0.008	−0.038	+	=	Spatial adaptation and thermal conformance
*Trypocopris pyrenaeus* (Charpentier, 1825)	283	G	T	86.3	0.18	0.000	0.031 Y	10.131	0.032	−0.030	+	=	Spatial adaptation and thermal conformance
*Caccobius schreberi* (Linnaeus, 1758)	574	S	H	2.6	−0.38	0.002	−0.011	0.347 Y	0.011	−0.005	+	=	Spatial adaptation and thermal conformance
*Euoniticellus fulvus* (Goeze, 1777)	899	S	H	15.9	−0.12	0.000	0.007	7.288 Y	0.026	−0.009	+	=	Spatial adaptation and thermal conformance
*Euonthophagus gibbosus* (Scriba, 1790)	224	S	H	13.1	−0.14	−0.063 Y	−0.014	11.660 Y	−0.006	−0.032	+	=	Spatial adaptation and thermal conformance
*Onthophagus fracticornis* (Preyssler, 1790)	388	S	H	13.1	−0.39	−0.014	−0.045	0.795 Y	−0.025	−0.031	+	=	Spatial adaptation and thermal conformance
*Onthophagus opacicollis* Reitter, 1893	441	S	H	5.4	0.09	0.025 Y	0.016	9.431 Y	0.015	−0.032	+	=	Spatial adaptation and thermal conformance
*Onthophagus punctatus* (Illiger, 1803)	424	S	H	3.7	−0.03	−0.028 Y	−0.059	8.459 Y	0.013	−0.027	+	=	Spatial adaptation and thermal conformance
*Onthophagus vacca* (Linnaeus, 1767)	1034	S	H	22.5	−0.17	−0.003	0.025 Y	7.010	0.011	−0.020	+	=	Spatial adaptation and thermal conformance
*Agrilinus constans* (Duftschmid, 1805)	530	A	E	3.1	0.21	0.014 Y	0.027 Y	2.441	0.056 Y	0.037 Y	+	+	spatial adaptation & thermal tolerance
*Aphodius coniugatus* (Panzer, 1795)	366	A	E	19.0	0.07	0.014	0.036 Y	3.309	0.121 Y	0.089 Y	+	+	spatial adaptation & thermal tolerance
*Biralus satellitius* (Herbst, 1789)	443	A	E	6.1	0.10	−0.015	0.057 Y	1.573	0.061 Y	0.028 Y	+	+	spatial adaptation & thermal tolerance
*Colobopterus erraticus* (Linnaeus, 1758)	1276	A	H	4.1	0.21	−0.008	0.006	1.021 Y	0.055 Y	0.029 Y	+	+	spatial adaptation & thermal tolerance
*Coprimorphus scrutator* (Herbst, 1789)	853	A	E	47.1	0.03	0.019 Y	0.044 Y	2.242	0.038 Y	0.021 Y	+	+	spatial adaptation & thermal tolerance
*Euorodalus coenosus* (Panzer, 1798)	273	A	E	1.3	0.18	−0.011	0.022 Y	10.134 Y	0.101 Y	0.040 Y	+	+	spatial adaptation & thermal tolerance
*Otophorus haemorrhoidalis* (Linnaeus, 1758)	1167	A	E	1.6	0.03	0.002	0.015 Y	4.153	0.046 Y	0.016 Y	+	+	spatial adaptation & thermal tolerance
*Agolius bonvouloiri* (Harold, 1860)	259	A	E	10.7	0.07	−0.004	−0.003	−0.997 Y	0.033	0.018 Y	−	+	spatial discordance & thermal tolerance
*Calamosternus granarius* (Linnaeus, 1767)	926	A	E	1.6	−0.50	−0.016 Y	−0.001	5.358	0.025 Y	−0.010	−	+	spatial discordance & thermal tolerance
*Melinopterus consputus* (Creutzer, 1799)	312	A	E	1.1	0.02	−0.052 Y	0.059 Y	−4.900 Y	0.195 Y	0.105 Y	−	+	spatial discordance & thermal tolerance
*Pleurophorus caesus* (Creutzer, 1796)	133	A	E	0.4	0.02	−0.070 Y	−0.059 Y	18.254	0.232 Y	0.084	−	+	spatial discordance & thermal tolerance
*Rhodaphodius foetens* (Fabricius, 1787)	126	A	E	6.9	0.09	−0.001	−0.048 Y	−9.094 Y	0.073	0.085 Y	−	+	spatial discordance & thermal tolerance
*Ceratophyus hoffmannseggi* Fairmaire, 1856	203	G	H	469.4	0.13	−0.076 Y	−0.037	−20.612 Y	0.016	0.172 Y	−	+	spatial discordance & thermal tolerance
*Silphotrupes escorialensis* (Jekel, 1866)	198	G	H	46.5	0.07	0.006	−0.020 Y	−1.081 Y	0.021	0.014 Y	−	+	spatial discordance & thermal tolerance
*Onthophagus furcatus* (Fabricius, 1781)	982	S	H	2.5	−0.46	−0.003	−0.027 Y	4.987	0.032 Y	−0.002 Y	−	+	spatial discordance & thermal tolerance
*Onthophagus merdarius* Chevrolat, 1865	218	S	H	7.6	0.08	−0.027	0.048 Y	−14.956 Y	0.093 Y	0.098 Y	−	+	spatial discordance & thermal tolerance
*Onthophagus ovatus* (Linnaeus, 1767)	116	S	H	2.3	−0.03	−0.010	−0.050 Y	7.143	0.073 Y	0.028	−	+	spatial discordance & thermal tolerance
*Aphodius foetidus* (Herbst, 1783)	1106	A	E	5.4	0.31	0.000	0.001	7.382	0.054 Y	0.012 Y	=	+	spatial conformance & thermal tolerance
*Bodilopsis rufa* (Moll, 1782)	575	A	E	4.1	0.08	−0.007	−0.010	6.291	0.059 Y	0.028 Y	=	+	spatial conformance & thermal tolerance
*Calamosternus mayeri* (Pilleri, 1953)	179	A	E	0.9	0.01	0.029	0.016	2.092	0.192 Y	0.091	=	+	spatial conformance & thermal tolerance
*Teuchestes fossor* (Linnaeus 1758)	975	A	H	30.8	0.11	−0.004	−0.008	3.121	0.046 Y	0.021 Y	=	+	spatial conformance & thermal tolerance
*Onthophagus latigena* d'Orbigni, 1897	400	S	H	2.2	0.03	−0.048	0.017	−2.293	0.136 Y	0.065	=	+	spatial conformance & thermal tolerance
